# Editorial: Univentricular Heart

**DOI:** 10.3389/fped.2015.00075

**Published:** 2015-09-14

**Authors:** Antonio F. Corno

**Affiliations:** ^1^East Midlands Congenital Heart Centre, Glenfield Hospital, Leicester, UK

**Keywords:** bidirectional Glenn, congenital heart surgery, Fontan, palliation, univentricular heart

## Introduction

Decades ago, the surgeons were asking themselves if it was possible to improve the natural history of patients with “functionally” univentricular heart (1). In order to facilitate the understanding and the subsequent decision making, for the first time, the patients with “functionally” univentricular heart were divided into two groups: (1) restricted pulmonary blood flow and (2) unrestricted pulmonary blood flow, with or without obstruction to the systemic blood flow (1).

Since the first stage in both groups is a palliative approach, over the years, the progress with the available materials and technology facilitated the surgical palliations to increase the pulmonary blood flow with systemic-to-pulmonary shunt (modified Blalock–Taussig shunt) (2, 3) as well as the control of distal pulmonary artery pressure and flow with adjustable pulmonary artery banding (4, 5).

The introduction of the use of the end-to-side anastomosis of the transected main pulmonary artery to the ascending aorta (Damus–Kaye–Stansel), as source of systemic blood flow, facilitated the management of patients with “functionally” univentricular heart with obstruction to the systemic blood flow (6).

The results of the surgical management have been substantially improved by the introduction of the bidirectional Glenn (end-to-side anastomosis of the superior vena cava to the right pulmonary artery) as second stage between the first palliation to control the pulmonary blood flow and the final stage of dividing the systemic and the pulmonary venous returns (7).

The introduction of the extracardiac modified Fontan procedure as third surgical stage contributed to provide adequate early (8) and late (9) results, with better survival and lower incidence of complications.

The improved knowledge of the appropriate timing of indication and the adequate preparation for the third stage with Fontan circulation (10) together with progress in the intraoperative management (11) provided a further improvement in the surgical outcomes.

The availability of adequate research laboratories allowed the performance of sophisticated experimental studies on animals to improve the available knowledge on the mechanisms of chronic hypoxia (12, 13) and pulmonary hypertension (14, 15), characterizing respectively the two groups of patients, with “functionally” univentricular heart and restrictive or unrestrictive pulmonary blood flow.

Finally, the combination of experimental research with computational fluid dynamic studies prompted to evaluate the feasibility of the mechanical assistance for patients at high risk for modified Fontan procedure or with failure of the Fontan circulation (16).

## Recent Literature

Several aspects of the management of patients with “functionally” univentricular heart have been investigated in the recent literature.

Considering the peculiar pathophysiology with difficult balance between systemic and pulmonary blood flow, often these patients require a period of stabilization before any surgical step, sometimes including resuscitation because of cardiac arrest or severe metabolic derangement (17).

With regard to the ventricular morphology, several studies are still investigating the long-term ventricular function to evaluate if there is a difference in the performance between the right and left ventricular morphology (18).

Some brave surgeons, working in emergent economies, decided to proceed with a pulmonary artery banding as first palliative stage in patients with “functionally” univentricular heart at an average age much older than for the conventional management. Contrary to the expectations and the available knowledge, they have reported quite good results, with adequate long-term follow-up after the subsequent surgical steps (19).

Since the final goal of the multistage surgical procedures in patients with “functionally” univentricular heart is the Fontan circulation, one of the main factors allowing to reach the target is the adequate growth of the pulmonary arteries. Several studies have investigated the potential growth of the pulmonary arteries between the first palliation (either a modified Blalock–Taussig shunt or a pulmonary artery banding) and the bidirectional Glenn, and between the bidirectional Glenn and the modified Fontan procedure, using different criteria to evaluate the size of the pulmonary arteries: *Z* score, McGoon ratio, and Nakata index. Disagreement still exists about the growth of the pulmonary arteries following interstage procedures (20).

The most challenging group of patients with “functionally” univentricular heart is the group with heterotaxy because of the frequently associated anomalies of the systemic and venous connections, the variable anatomy of the sinus node and conduction system, the common presence of atrioventricular valve regurgitation, and the difficulty in obtaining with surgical palliations an adequate balance between the systemic and the pulmonary blood flow (21).

With regard to the preservation of the ventricular function for the long-term performance of the unique ventricular chamber available, in association with the best possible choice of timing and type of the surgical procedures, attempts have been done at improving the ventricular function with medical treatments potentially unloading the single ventricle (22).

Thanks to the improved surgical results obtained in the last decades with relatively reduced mortality and morbidity, in the last few years, the attention of the caregivers has concentrated on the quality of life and the neurodevelopmental and cognitive development following the surgical procedures (23).

The goal in patients with “functionally” univentricular heart is always to possibly reach the stage of Fontan circulation, and the recent literature has recently reported articles on the importance of imaging in the perioperative assessment of Fontan patients (24), on the results of extracardiac Fontan procedure in adult patients (25), on the feasibility of establishing the Fontan circulation without cardiopulmonary bypass (26), on the post-operative respiratory function (27), on the not infrequent need for conversion of the previous Fontan procedure associated with arrhythmias surgery (28), and on the need of a multidisciplinary team of specialists for the long-term follow-up of the patients after Fontan type of surgery (29).

More importantly, the data collected in the North-American and European Congenital Data Base allow a very extensive documentation of the currently available information on indication, clinical practice, and outcomes in this challenging group of patients (30).

Despite the fact that in patients with “functionally” univentricular heart almost all surgeons are planning surgical steps toward the accomplishment of the Fontan circulation, few surgeons are still offering a ventricular septation in selected cases with double inlet left ventricle, with decent long-term outcomes (31).

Nevertheless, in a not negligible percentage of patients, because of the failure of the conventional surgery, heart transplant has to be considered in the presence of end-stage circulatory failure after Fontan procedure (32, 33).

## Research Topic

Since there are still many unsolved questions and issues in the management of patients with “functionally” univentricular heart, this Research Topic has been organized to stimulate the people involved with these problems to provide their contribution to this timely and difficult matter.

The patients with “functionally” univentricular heart have been taken into consideration from the moment of pre-natal diagnosis (34) through detailed analysis of the complex and variable underlying morphology (35).

The decision-making process has been extensively investigated from the first surgical stage until the third stage (36), and particular efforts have been dedicated to the unsolved issues in the specific group of patients with hypoplastic left heart syndrome (37), including the potential contribution given by the computational fluid dynamic studies (38).

Finally, the long history of the surgical approaches used to separate the pulmonary from the systemic venous return obtaining the Fontan circulation has been reviewed, with precise analysis of the advantages and disadvantages of all the surgical techniques (39).

The extensive review and update performed in this Research Topic will be hopefully useful to all the readers involved with the treatment of this very difficult group of patients.

## Conflict of Interest Statement

The author declares that the research was conducted in the absence of any commercial or financial relationships that could be construed as a potential conflict of interest.
